# E-Cigarette Use in Young Adult Never Cigarette Smokers with Disabilities: Results from the Behavioral Risk Factor Surveillance System Survey

**DOI:** 10.3390/ijerph18105476

**Published:** 2021-05-20

**Authors:** Nkiruka C. Atuegwu, Mark D. Litt, Suchitra Krishnan-Sarin, Reinhard C. Laubenbacher, Mario F. Perez, Eric M. Mortensen

**Affiliations:** 1 Department of Medicine, University of Connecticut School of Medicine, Farmington, CT 06030, USA; maperez@uchc.edu (M.F.P.); mortensen@uchc.edu (E.M.M.); 2 Division of Behavioral Sciences and Community Health, University of Connecticut School of Medicine, Farmington, CT 06030, USA; litt@uchc.edu; 3 Department of Psychiatry, Yale University School of Medicine, New Haven, CT 06519, USA; suchitra.krishnan-sarin@yale.edu; 4 Laboratory for Systems Medicine, Department of Medicine, University of Florida, Gainesville, FL 32610, USA; Reinhard.Laubenbacher@medicine.ufl.edu

**Keywords:** young adults, ENDS, e-cigarette, disability, vaping, electronic nicotine delivery system, LASSO, BRFSS, machine learning, never-smokers

## Abstract

Young adult never cigarette smokers with disabilities may be at particular risk for adopting e-cigarettes, but little attention has been paid to these people. This study examines the associations between different types of disability and e-cigarette use in this population. Young adult never-smokers from the 2016–2017 Behavioral Risk Factor Surveillance System (BRFSS) survey who were either never or current e-cigarette users (*n* = 79,177) were selected for the analysis. The Least Absolute Shrinkage and Selection Operator (LASSO) algorithm was used to select confounders for multivariable logistic regression models. Multivariable logistic regression models were used to determine the associations between current e-cigarette use and different types of disability after incorporating BRFSS survey design and adjusting for confounders. Young adult never-smokers who reported any disability had increased odds (OR 1.44, 95% CI 1.18–1.76) of e-cigarette use compared to those who reported no disability. Young adult never-smokers who reported self-care, cognitive, vision, and independent living disabilities had higher odds of e-cigarette use compared to those who reported no disability. There was no statistically significant difference in the odds of e-cigarette use for those reporting hearing and mobility disabilities compared to those who reported no disability. This study highlights the need for increased public education and cessation programs for this population.

## 1. Introduction

In recent years, the use of electronic nicotine delivery systems, also known as e-cigarettes, has increased in the U.S., especially in youth and young adults [1]. Young adults are more likely to be current e-cigarette users compared to older adults, and a significant percentage of young adults who are never cigarette smokers use e-cigarettes [2,3]. In 2018–2019, 23% of adult current e-cigarette users were never cigarette smokers and 87% of e-cigarette users who were never-smokers were less than 35 years old [3]. E-cigarettes contain potentially harmful toxicants and e-cigarette use may be associated with asthma, COPD, respiratory symptoms, prediabetes, oral diseases and depression [4,5,6,7,8,9]. The use of e-cigarettes may also increase the likelihood of future cigarette smoking, marijuana and non-prescribed drug use [10,11,12].

In 2016, 25.7% of U.S. adults, an estimated 61.4 million people, reported a disability. The most prevalent types of disability were self-care (3.7%), vision (4.6%), independent living (6.8%), cognitive (10.8%), and mobility (13.7%) [13]. Adults who report disabilities are more likely to have ever smoked and currently smoke cigarettes [14], possibly in an effort to cope with the added stress of the disability itself [15]. In 2017, college students who reported any disability had a higher prevalence of tobacco (cigarettes, cigars, pipes, and smokeless tobacco) use compared to those without any disability, and were nearly twice as likely to meet the criteria for past-month nicotine dependence [16]. The published evidence of disability-related differences in tobacco use is mostly limited to cigarette and other tobacco product use, and there is little research on e-cigarette use in young adults with disabilities.

Increased use of e-cigarettes has been reported in adults with a history of mental health conditions [17,18]. Du et al. showed that adult females who report a disability had increased odds of ever using e-cigarettes compared to females without a disability [19]. Gimm et al. reported that working age (18–64 years) adults who reported disabilities were more likely to use e-cigarettes compared to adults who did not report a disability [20]. However, what holds for older adults may not hold for younger adults. This is because the reasons for e-cigarette use varies between younger and older adults and varies based on cigarette smoking status [21,22]. Thus, we still know very little about the current use of e-cigarettes in young adult never cigarette smokers who report disabilities.

Our analysis focuses on young adult (<35 years old) never cigarette smokers who are current e-cigarette users for several reasons. The use of e-cigarettes is rising among never cigarette smokers and most never-smokers who use e-cigarettes are less than 35 years old [3]. Although some studies have shown that e-cigarettes may contain fewer toxicants than cigarettes, never-smokers are exposed to toxicants in e-cigarettes and the long term effects of these toxicants on health are largely unknown [23]. Furthermore, pod-based styles of e-cigarettes, which are commonly used by e-cigarette users, may contain as much nicotine as one pack of cigarettes [24]. Nicotine affects the prefrontal cortex, which is involved in cognitive development, executive function, and inhibitory control and is not fully matured until the mid-twenties [25]. Focusing on young adult never-smokers who are most at risk for the potential adverse health effects of e-cigarette use is desirable because we may be able to intervene before they become more stable in their habits. Young adults who are able to stop using nicotine and tobacco products in their 20s and early 30s are less likely to resume these products as older adults [26]. Identifying particularly vulnerable subgroups of young adults is therefore even more important.

This study built on recent work by Atuegwu et al. that used machine learning (ML) techniques to identify factors associated with current e-cigarette use in young adult never cigarette smokers. The ML algorithms identified previously unreported potential associations between e-cigarette use and some types of disability in young adult never cigarette smokers [27]. The goal of this study was to examine the associations between the different types of disability and e-cigarette use in young adult never cigarette smokers. This is important because people with disabilities experience poorer health and functioning and higher all-cause mortality rates compared with people without disabilities [28].

## 2. Materials and Methods

### 2.1. Data

The 2016 and 2017 Behavioral Risk Factor Surveillance System (BRFSS) survey data were combined and used for the study. Two years of BRFSS survey data were combined in order to increase the sample size used for the study. The BRFSS survey was designed such that data from multiple years could be combined. Details as to how to combine data from multiple years for analysis can be found elsewhere [29,30].

The BRFSS survey is a system of ongoing, self-reported cellular and landline telephone-based surveys that is designed to collect uniform state-specific data on the use of preventive health services, healthcare access, chronic diseases, health conditions, risk behaviors and demographics from noninstitutionalized adults (≥18years) living in the United States and participating territories. The survey is administered and supported by the Centers for Disease Control and Prevention (CDC) and it is a collaborative effort between all of the states in the United States, participating territories and the CDC. The survey consists of core questions used by every state and participating territory, and optional questions that are included by some states and participating territories. For the cellular telephone survey, data is collected from adults living in private residences or college housing and for the landline survey, data is collected from a randomly selected adult in a household. Landline numbers for the survey are randomly selected using a disproportionate stratified sample design, and cellphone numbers for the survey are randomly selected using cellphone sampling frames. The BRFSS data weights, which incorporate the characteristics of the population and the BRFSS complex design, are used to make the sample data more representative of the noninstitutionalized adults in the US and participating territories. Details on the BRFSS design can be found elsewhere [31,32]

### 2.2. Disability Types and Status

Different types of disability were assessed in the BRFSS with the following questions as recommended by the United States Department of Health and Human Services [33]:

1. Vision disability: “Are you blind, or do you have serious difficulty seeing, even when wearing glasses?”
2. Hearing disability: “Are you deaf, or do you have serious difficulty hearing?”
3. Cognitive disability: “Because of a physical, mental, or emotional condition, do you have serious difficulty concentrating, remembering, or making decisions?”
4. Mobility disability: “Do you have serious difficulty walking or climbing stairs?”
5. Independent living disability: “Because of a physical, mental, or emotional condition, do you have difficulty doing errands alone such as visiting a doctor’s office or shopping?”
6. Self-care disability: “Do you have difficulty dressing or bathing?”

Participants who answered “yes” to a type of disability were considered as having that disability. Participants who answered “no” to all the types of disability were defined as having no disability. Participants could report more than one type of disability.

### 2.3. Study Sample

Participants who were 18 to 34 years old, were never cigarette smokers and were current or never e-cigarette users were included in the analysis. Participants were never e-cigarette users if they answered “no” to the question “Have you ever used an e-cigarette or other electronic vaping product, even just one time, in your entire life?” Participants were current e-cigarette users if they reported currently using e-cigarette or other electronic vaping products every day or some days. Participants were never cigarette smokers if they reported smoking fewer than 100 cigarettes in their entire life. A total of 79,539 participants who met the inclusion criteria were included in the analysis.

### 2.4. Data Preprocessing

Those BRFSS core questions that were the same in both the 2016 and 2017 surveys were used for the analysis. Core survey questions include questions on current health-related perceptions, conditions, and behaviors, healthcare access, alcohol and tobacco use (cigarettes, e-cigarettes and smokeless tobacco), as well as demographic questions. Details about the core survey questions can be found elsewhere [34,35]. To preprocess the data variables with no research implications (e.g., interview month), variables not related to demographics, behaviors, and health, or variables that were used to create other variables were removed. Missing survey data that could be determined from other survey questions, such as questions that were skipped because of previous response to another question, were changed to an appropriate value. Categorical variables to which participants responded “Refused/not sure/don’t know/missing” were assigned a new categorical value. After preprocessing the data, 40 potential confounder variables were identified.

### 2.5. Statistical Analysis

#### 2.5.1. Confounder Selection

Confounding is a significant limitation to making inferences with observational data such as the BRFSS survey data. Creating statistical models that adjust for confounding variables can reduce this limitation [36]. This requires that confounding variables are identified. However, omitting important confounding variables can bias the results of the analysis [37]. A way to reduce this bias is through the use of a machine learning algorithm to automatically select confounders for the multivariable logistic regression models. This reduces the possibility of missing important cofounders and tailors the confounders to each population of interest. For this study, the Least Absolute Shrinkage and Selection Operator (LASSO) algorithm [38,39], a machine learning algorithm that can be used for variable selection, was used [39,40,41].

The LASSO algorithm has been used to select variables associated with current e-cigarette use in young adult never cigarette smokers [27] and has been used in other types of survey and medical data [42,43,44]. The LASSO algorithm penalizes the regression model parameters and reduces the least important variables to zero, thereby selecting the most important variables for the model [39]. For each type of disability, participants who reported the disability of interest and participants who reported no disability were used to create subgroups for analysis. For each subgroup, two sets of LASSO analyses were performed. The first was used to select features associated with the disability of interest and the second was used to select the features associated with e-cigarette use in that same subgroup. Variables that were associated with both the disability of interest and e-cigarette use for the different disability subgroups were selected as potential confounders. This was based on the definition of cofounders [45].

To reduce the limitations and errors due to one application of the LASSO algorithm, and to reduce the sensitivity of LASSO to small perturbations in the data [46], 300 iterations of LASSO were performed. For each iteration, random samples comprising of 80% of the original data were used [27]. For each iteration, a 10-fold cross-validation selected the lambda ($\lambda_{m}$) that produced the minimum mean cross validation error [39,47]. For each iteration, variables with coefficients that were not equal to zero for categories other than “Refused/not sure/don’t know/missing” for $\lambda_{m}$ were selected. All the variables that were selected in >95% of the iterations were considered significant variables for e-cigarette use or the disability of interest. More details can be found elsewhere [27].

Table 1 shows the variables that were selected by the LASSO algorithm as significantly associated with both the different types of disability and e-cigarette use. The superscripts 1–6 indicate the type of disability the variable acts as a confounder for in the analysis. Table 1 also shows the weighted descriptive statistics of the confounders used for the multivariable logistic regression models. For example, being a veteran was associated with both e-cigarette use and mobility disability, therefore, it was considered a confounder for mobility disability.

Among the confounders, modifiable characteristics such as employment status, number of days of poor physical health and mental health in past 30 days, history of depressive disorders and skipping medical care in the past 12 months due to cost were always chosen by the LASSO algorithm to be associated with all the different types of disability and e-cigarette use. Age, race/ethnicity and gender, which are considered non-modifiable demographic exposures [48], were included in all analyses as confounders.

#### 2.5.2. Multivariable Logistic Regression

To account for the potential bias that can occur if the indicator method or list wise deletion are used when creating the multivariable logistic regression models, missing data in which participants reported “refused/not sure/don’t know/missing” were imputed using Multiple Imputation by Chained Equations (MICE) [49]. Only the categorical variables contained missing data and these were imputed using polytomous regression for more than two categories and logistic regression for binary data [49]. All covariates selected by LASSO for the different types of disabilities (shown in Table 1) as well as the outcome variables were included in the imputation process. Fifty imputed data sets were generated and 30 iterations were used for each imputation in order to ensure convergence. 

For each disability of interest we built two multivariable logistic regression models. The first model adjusted only for age, race/ethnicity and gender, which are non-modifiable demographic exposures. The second model adjusted both for non-modifiable demographic exposures and the confounders identified by the LASSO algorithm (denoted in Table 1 with a superscript). For each multivariable logistic regression model, we used the generalized variance-inflation factors (GVIF), which are more appropriate for regressions with categorical variables, to detect multicollinearity [50]. To make the GVIFs comparable across dimensions, we calculated $GVIF^{\frac{1}{2*df}}$. where df is the degrees of freedom of the variables [50]. For all the multivariable logistic regression models, $GVIF^{\frac{1}{2*df}}$ was ≤ 1.4 which is less than the threshold suggested for high collinearity [51]. The BRFSS survey complex design was included in the analysis, to adjust for nonresponse bias, probability of selection and non-coverage errors. The survey weights that account for the combined data from the 2016 and 2017 BRFSS survey were calculated as detailed in the report on BRFSS analysis from the CDC [29,30]. Multivariable logistic regression models were created on each simulated complete dataset from the multiple imputations and the results were combined to obtain the odds ratios and confidence intervals that incorporate missing-data uncertainty [52]. These results are shown in Table 2. R version 3.6.1 [53] was used for the analyses.

## 3. Results

Among young adult never-smokers(*n* = 79,539) who reported never and current e-cigarette use, 44.8% (95% CI 44.2–45.5) were males, 11.9% (95% CI 11.5–12.3) reported any disability and 4.4% (95% CI 4.2–4.7) were current e-cigarette users. The prevalences of the different types of disability were 7.6% (95% CI 7.3–7.9) for cognitive, 2.8% (95% CI 2.5–3.0) for independent living, 2.3% (95% CI 2.2–2.5) for mobility, 2.1% (95% CI 1.9–2.3) for vision, 1.3% (95% CI 1.2–1.5) for hearing, and 0.8% (95% CI 0.7–0.9) for self-care. Among young adult never-smokers who reported any type of disability, 7.7% (95% CI 6.7–8.7) were current e-cigarette users.

After adjusting for only non-modifiable demographic exposures (age, sex and race/ethnicity), participants who reported any disability, vision, cognitive, independent living or self-care disabilities had increased odds of e-cigarette use compared to those who reported no disability (Model 1 in Table 2). For the expanded models that were adjusted for non-modifiable demographic exposures and the confounders specific to each type of disability shown in Table 1, young adult never-smokers who reported any disability had increased odds of current e-cigarette use (OR 1.44, 95% CI 1.18–1.76) compared to those who reported no disability. Young adult never-smokers who reported self-care (OR 2.37, 95% CI 1.38–4.06), independent living (OR 1.75, 95% CI 1.27–2.42), cognitive (OR 1.58, 95% CI 1.25–2.01) and vision (OR 1.48, 95% CI 1.02–2.15) disabilities had increased odds of e-cigarette use compared to those who reported no disability. Hearing (OR 0.90, 95% CI 0.58–1.40) and mobility disabilities (OR 1.09, 95% CI 0.76–1.57) were not statistically significantly associated with current e-cigarette use (Model 2 in Table 2).

**Table 2:** Results from the multivariable logistic regression models used to determine the association between current e-cigarette use and the different types of disabilities. Odds ratios and confidence intervals are the combined results from each simulated complete dataset from the multiple imputations. The reference group is young adult never cigarette smokers who report no disability. Sample size is the mean sample size from all the multiple imputed datasets.

Disability Type	Model 1 ^1^	Model 2 ^2^
	OR (95 % CI) ^2^	OR (95 % CI)
Any (*n* = 79,539)	2.05 (1.75–2.41)	1.44 (1.18–1.76)
Vision (*n* = 71,937)	1.99 (1.43–2.79)	1.48 (1.02 -2.15)
Hearing (*n* = 71,376)	1.21 (0.81–1.81)	0.90 (0.58–1.40)
Cognitive (*n* = 76,053)	2.38 (1.98–2.86)	1.58 (1.25–2.01)
Mobility (*n* = 72,101)	1.59 (1.10–2.29)	1.09 (0.76–1.57)
Independent Living (*n* = 72,400)	2.63 (1.98–3.50)	1.75 (1.27–2.42)
Self-care (*n* = 70,861)	2.95 (1.70–5.12)	2.37 (1.38–4.06)

^1^ Adjusted for age, race/ethnicity and gender; ^2^ Adjusted for age, race/ethnicity and gender and the confounders identified by LASSO in Table 1.

## 4. Discussion

Our findings show that young adult never-smokers who report independent living, cognitive, self-care and vision disabilities have higher odds of e-cigarette use compared to those who report no disability. This is concerning, because the use of e-cigarettes in never-smokers can lead to nicotine dependence and other adverse outcomes. Interventions to reduce the use of e-cigarettes in young adult never cigarette smokers with disabilities could be targeted more effectively to these subgroups.

Our results are consistent with some of the findings on the use of e-cigarettes in working age adults (<65 years) with disabilities. Gimm et al. found higher odds of e-cigarette use in working age adults who reported cognitive, independent and mobility disabilities compared to those who reported no disability. They also found lower odds of e-cigarette use in working age adults who reported hearing disability compared to those who reported no disability [20]. We found increased odds of e-cigarette use in young adult never cigarette smokers who reported cognitive and independent living disabilities, and we also found increased odds of e-cigarette use in participants who reported vision and self-care disabilities compared to those who reported no disability. We found no statistically significant association between mobility and hearing disabilities and current e-cigarette use in young adult never cigarette smokers in our fully adjusted models, although we found an association between mobility disability and current e-cigarette use in the model that adjusted for only non-modifiable demographic variables. These differences may be due to the different subgroups of the U.S. population that we studied. We focused on young adult never-smokers and studies have shown that the prevalence of the different types of disabilities varies based on age [20], and the prevalence and reasons for e-cigarette use varies based on cigarette smoking status and age [21,22]. These differences may account for the differences in the odds ratios of e-cigarette use in both studies. Future work is needed to understand and examine these differences.

Physicians and caregivers need to be aware of the increased potential of e-cigarette use in young adults with disabilities and should routinely screen for this in order to provide effective cessation counseling for this population. People with disabilities are often excluded from participation in mainstream public health services and programs and the inclusion of young adults with disabilities in effective mainstream public health programs will improve the health of this vulnerable population [54]. These findings can help inform policy makers and guide the development of e-cigarette cessation interventions that are targeted towards or can accommodate, young adults with disabilities. Tailoring tobacco cessation programs to the characteristics unique to people with disabilities can provide effective cessation interventions among this population [55]. These interventions should be designed in partnership with the disability community and should employ accommodations that support the participation of young adults with disabilities in the program’s activities [56]. Some examples include the modification of existing e-cigarette cessation interventions for the general public. For example, printed information on e-cigarette cessation services could be modified to be in an accessible format (e.g., large print) for young adults with visual disabilities and designed at a level that can be accessible to young adults with cognitive disabilities. 

The current study also validated the findings on e-cigarette use and disabilities from our previous work. Previously, we used two ML algorithms (Boruta and LASSO) to select features that were potentially associated with current e-cigarette use in young adult never cigarette smokers. The ML approach identified previously unreported features associated with e-cigarette use such as cognitive, independent living, vision, and self-care disabilities. The ML approach found no association between e-cigarette use and hearing and mobility disabilities [27]. In this study, these findings were confirmed after creating subgroups with participants who reported different disabilities and no disability, and adjusting with confounders specific to the disability of interest.

We can speculate on the attraction of e-cigarettes to individuals with disabilities. As with older adults, young adults with disabilities may use e-cigarettes to cope with daily stressors that they experience. Nicotine, which is the primary constituent of most e-cigarettes, triggers the release of neurotransmitters which reduce stress and anxiety, and induce pleasure [57]. People with disabilities were more likely to feel worried, tense, and anxious for more than half of the previous month, compared to those without disabilities [58]. While studies of causality, i.e., whether stressors lead to e-cigarette use or whether e-cigarette use leads to stress, still remain to be conducted, existing evidence does suggest that e-cigarette use is associated with higher levels of psychological distress [59]. E-cigarette use in individuals with disabilities may also be related to depression and poor mental health. People with disabilities are more likely to report depression, poor mental health and frequent mental distress compared to those without a disability [60]. Obisesan et al. found that current e-cigarette users were more likely to report a history of depression and more than one day of poor mental health compared to never e-cigarette users [8].

Strengths of this study include the use of a representative sample of U.S. young adult never cigarette smokers who were current or never e-cigarette users. Additionally, the use of a ML approach to select confounders reduces the possibility of omitting important and previously unknown confounders and also tailors the cofounders to the different types of disability, which can reduce overfitting of the models. Furthermore, this ML approach may be used to select confounders for models as the dimensionality of the data increases. The LASSO algorithm was able to select some factors associated with disabilities such as asthma, HIV risky behavior, depression disorder, skipping medical care in the past 12 months due to cost and poor mental and physical health in the past 30 days [60,61,62,63]. Additionally, the LASSO algorithm was also able to select factors that were associated with e-cigarette use [2,27].

Some limitations include the lack of biochemical confirmation of conventional cigarette use and e-cigarette use and the lack of medical confirmation of disabilities. These may lead to over-reporting or underreporting of e-cigarette use, cigarette use and disabilities, which may bias the results. Additionally, the data is self-reported, and this can lead to recall and diagnosis misclassification bias. The data is cross-sectional and thus we cannot establish causality between disability and e-cigarette use. Additionally, the survey has no information on the types of e-cigarettes used, or the duration and quantity of e-cigarettes used. Therefore, we cannot estimate the severity of e-cigarette use in this population. The survey has no information on the underlying medical condition, duration or permanence of the disability, so it not possible to differentiate e-cigarette use by the cause and duration of the disability. The data is unbalanced and this can affect the identification of some confounders associated with disabilities and e-cigarette use. Some of the confounders identified by LASSO may have been caused by disability and as such were not truly confounders in the model. Finally, the BRFSS surveys the noninstitutionalized U.S. population and as such, our results may not be representative of all young adult never-smokers with disabilities, especially those living in institutions or congregate care settings.

## 5. Conclusions

The study shows that young adult never-smokers who report disabilities are more likely to be current e-cigarette users compared to young adult never-smokers without disabilities. Additionally, young adult never-smokers who report self-care, cognitive, independent living and vision disabilities have higher odds of current e-cigarette use compared to those who report no disabilities. This highlights the need for effective public health and cessation interventions designed to accommodate young adults with disabilities, especially the subgroups with increased odds of e-cigarette use. Additional research is needed to study the health effects of e-cigarette use in young adult never-smokers with disabilities, the reasons for e-cigarette use in young adults with disabilities and the differences in e-cigarette use for the different types of disabilities. In addition, the associations between e-cigarette use and the permanence of the disability as well as the underlying medical reasons for the disability need to be studied.

## Figures and Tables

**Table 1:** Weighted descriptive statistics of the confounders selected by LASSO stratified by e-cigarette use. Superscripts (1–6) indicate the type of disability the variable is associated with and the type of disability the variable acts as a cofounder for in the analyses. Subscripts (7–13) describe some of the variables in detail.

Variables (*n* = Refused/Not Sure/Don’t Know/Missing)		Current E-Cigarette User *n* = 3146 (95% CI)	Never E-Cigarette User *n* = 76,393 (95% CI)
Age (mean) (*n* = 0) ^1,2,3,4,5,6,7^		22.1 (21.9–22.4)	25.8 (25.8–25.9)
Gender (*n* = 36) ^1,2,3,4,5,6,7^			
	Male	67.1 (64.4–69.8)	43.8 (43.2–44.4)
	Female	32.7 (30.0–35.4)	56.2 (55.5–56.8)
Race and ethnicity (*n* = 1043) ^1,2,3,4,5,6,7^			
	White only, Non-Hispanic	57.0 (54.0–59.9)	48.0 (47.4 - 48.7)
	Black only, Non-Hispanic	11.2 (9.2–13.1)	13.8 (13.4–14.2)
	Other race only, Non-Hispanic	8.9 (7.0–10.8)	10.5 (10.0–11.0)
	Multiracial, Non-Hispanic	2.4 (1.7–3.1)	1.5 (1.4–1.6)
	Hispanic	18.8 (16.3–21.3)	24.7 (24.1–25.3)
Marital Status (*n* = 485) ^1,2,4,5,6,7^			
	Married	10.1 (8.4–11.7)	31.0 (30.5–31.6)
	Not currently married ^8^	2.9 (2.0–3.8)	4.2 (3.9–4.4)
	Never married	77.0 (74.6–79.4)	56.3 (55.7–57.0)
	Member of an unmarried couple	9.5 (7.8–11.3)	7.8 (7.5–8.2)
Education level (*n* = 228) ^1,2,3,4,5,6^			
	Did not graduate high school	9.0 (7.3–10.7)	10.9 (10.4–11.4)
	Graduated high school	41.6 (38.7–44.5)	26.9 (26.3–27.4)
	Attended or graduated college or technical school	49.3 (46.4–52.2)	62.0 (61.3–62.6)
Employment (*n* = 888) ^1,2,3,4,5,6,7^			
	Self-employed or employed for wages	58.8 (55.9–61.7)	61.2 (60.5–61.8)
	Not currently employed ^9^	11.7 (9.9–13.6)	15.8 (15.3–16.3)
	Student	28.5 (25.8–31.1)	21.9 (21.3–22.5)
Income (*n* = 13,956) ^1,3,4^			
	Less than $25,000	21.9 (19.6–24.2)	24.7 (24.1–25.2)
	$25,000 to less than $50,000	20.8 (18.5–23.0)	20.0 (19.5–20.5)
	$ 50,000 or more	35.0 (32.2–37.8)	36.2 (35.6–36.8)
Rent or own a home (*n* = 590) ^1,4,5^			
	Own a home	29.7 (26.8–32.7)	40.4 (39.8–41.1)
	Rent or other arrangements	68.4 (65.4–71.5)	58.6 (58.0–59.3)
Veteran (*n* = 83) ^5^		5.1 (3.9–6.2)	4.3 (4.0–4.5)
Skipped medical care in the past 12 months due to cost (*n* = 205) ^1,2,3,4,5,6,7^		14.2 (12.3–16.1)	12.9 (12.5–13.4)
Length of time since last routine checkup (*n* = 1696) ^1,2,4,6,7^			
	Less than 2 years	77.8 (75.3–80.4)	78.0 (77.5–78.5)
	Greater than 2 years but less than 5 years	12.0 (9.9–14.0)	10.7 (10.3–11.1)
	5 or more years ago or never	8.0 (6.3–9.7)	9.3 (8.9–9.7)
Days of poor physical health in past 30 days (*n* = 997) ^1,2,3,4,5,6,7^			
	0	61.8 (59.0–64.5)	69.4 (68.8–70.0)
	1–13	30.7 (28.1–33.3)	24.7 (24.2–25.3)
	14+	6.1 (4.9–7.3)	4.6 (4.3–4.9)
Days of poor mental health in past 30 days (*n* = 866) ^1,2,3,4,5,6,7^			
	0	44.4 (41.4–47.3)	59.5 (58.9–60.2)
	1–13	36.2 (33.4–38.9)	29.8 (29.2–30.4)
	14+	18.5 (16.2–20.8)	9.5 (9.2–9.9)
Seatbelt Use (*n* = 3059) ^3^			
	Always	75.3 (72.7–77.8)	83.3 (82.8–83.8)
	Not always	20.6 (18.3–22.9)	12.5 (12.0–12.9)
Internet use in past 30 days (*n* = 84) ^2,3,5,6,7^		98.5 (97.9–99.0)	94.7 (94.4–95.0)
Flu vaccine in past 12 months (*n* = 3413) ^1,4,6^		26.7 (24.1–29.2)	31.4 (30.8–32.0)
History of pneumonia vaccine (*n* = 19,116) ^1,5,6^		28.1 (25.4–30.8)	19.6 (19.1–20.2)
One or more drinks in past 30 days (*n* = 1032) ^1,2,4,5,6,7^		68.0 (65.1–70.8)	47.9 (47.2–48.5)
Binge drinker (*n* = 1705) ^1,6,10^		36.6 (33.9–39.4)	15.9 (15.5–16.4)
Heavy drinkers (*n* = 1908) ^4,11^		9.3 (7.7–10.8)	3.2 (3.0–3.4)
Current smokeless tobacco user (*n* = 68) ^1,2,3,4,5^		7.0 (5.8–8.2)	2.1 1.9–2.3)
Ever had a HIV test (*n* = 5857) ^1^		32.8 (30.0–35.5)	35.4 (34.8–36.0)
HIV High Risk behavior (*n* = 4532) ^1,2,3,4,5,6,12^		23.6 (21.1–26.1)	7.4 (7.1–7.8)
History of depressive disorders (*n* = 377) ^1,2,3,4,5,6,7^		20.9 (18.8–23.1)	12.1 (11.7–12.5)
History of Asthma (*n* = 571) ^1,2,3,4,5,6^			
	Currently have asthma	10.4 (8.8–12.0)	8.2 (7.9–8.6)
	No longer have asthma	8.5 (6.6–10.3)	5.5 (5.3–5.8)
**Outcomes**			
One or more disability (*n* = 362) ^13^			
	Any disability	20.8 (18.3–23.2)	11.5 (11.1–11.9)
	No disability	78.4 (76.0–80.8)	88.0 (87.6 -88.4)
Vision disability (*n* = 80)		3.5 (2.5–4.5)	2.1 (1.9–2.3)
Hearing disability (*n*= 59)		1.4 (0.9–1.9)	1.3 (1.2–1.5)
Cognitive disability (*n* = 270)		15.8 (13.6–18.0)	7.2 (6.9–7.6)
Mobility disability (*n* = 44)		2.5 (1.7–3.3)	2.3 (2.1–2.5)
Independent living disability (*n* = 96)		5.7 (4.4–7.1)	2.6 (2.4–2.8)
Self-care disability (*n* = 30)		1.6 (0.8–2.3)	0.8 (0.6–0.9)

^1^ Associated with e-cigarette use and any disability. Confounders for the multivariable regression model with any disability; ^2^ Associated with e-cigarette use and vision disability. Confounders for the multivariable regression model with vision disability; ^3^ Associated with e-cigarette use and hearing disability. Confounders for the multivariable regression model with hearing disability; ^4^ Associated with e-cigarette use and cognitive disability. Confounders for the multivariable regression model with cognitive disability; ^5^ Associated with e-cigarette use and mobility disability. Confounders for the multivariable regression model with mobility disability; ^6^ Associated with e-cigarette use and independent living disability. Confounders for the multivariable regression model with independent living disability; ^7^ Associated with e-cigarette use and self-care disability. Confounders for the multivariable regression model with self-care disability; ^8^ Participants who are separated, widowed or divorced; ^9^ Participants who are homemakers, unable to work, out of work or retired; ^10^ 5 or more drinks for males and 4 or more drinks for females on an occasion in the past 30 days; ^11^ 14 or more drinks for males and 7 or more drinks for females every week; ^12^ Participant reported any of the following in the past year: received money or drugs for sex, treatment for STD or sexually transmitted disease, use of non-prescribed intravenous drug; ^13^ Participants with missing data for one or more disability answered “Refused/not sure/don’t know/missing” for one or more types of disability while also reporting no disability for the other types of disability.

## Data Availability

Publicly available datasets were analyzed in this study. This data can be found here: https://www.cdc.gov/brfss/annual_data/annual_2016.html (accessed on 19 May 2021) and https://www.cdc.gov/brfss/annual_data/annual_2017.html (accessed on 19 May 2021).
